# Radiation Dose in the Thyroid and the Thyroid Cancer Risk Attributable to CT Scans for Pediatric Patients in One General Hospital of China

**DOI:** 10.3390/ijerph110302793

**Published:** 2014-03-07

**Authors:** Yin-Ping Su, Hao-Wei Niu, Jun-Bo Chen, Ying-Hua Fu, Guo-Bing Xiao, Quan-Fu Sun

**Affiliations:** 1 Key Laboratory of Radiological Protection and Nuclear Emergency Chinese Center for Disease Control and Prevention, National Institute for Radiological Protection, Chinese Center for Disease Control and Prevention, Beijing 100088, China; E-Mails: yinpingsu@126.com (Y.-P.S.); crrw@nirp.cn (H.-W.N.); fuyinghua@nirp.cn (Y.-H.F.);; 2 Department of Imaging, The Second Yinzhou Hospital, Ningbo 315000, Zhejiang, China; E-Mail: nbchenjunbo@126.com; 3 Ningbo municipal agency for public health inspection, Ningbo 315000, Zhejiang, China; E-Mail: xiaogb@sohu.com

**Keywords:** cancer risk, pediatric CT, radiation dose

## Abstract

*Objective*: To quantify the radiation dose in the thyroid attributable to different CT scans and to estimate the thyroid cancer risk in pediatric patients. *Methods*: The information about pediatric patients who underwent CT scans was abstracted from the radiology information system in one general hospital between 1 January 2012 and 31 December 2012. The radiation doses were calculated using the ImPACT Patient Dosimetry Calculator and the lifetime attributable risk (LAR) of thyroid cancer incidence was estimated based on the National Academies Biologic Effects of Ionizing Radiation VII model. *Results*: The subjects comprised 922 children, 68% were males, and received 971 CT scans. The range of typical radiation dose to the thyroid was estimated to be 0.61–0.92 m*G*y for paranasal sinus CT scans, 1.10–2.45 m*G*y for head CT scans, and 2.63–5.76 m*G*y for chest CT scans. The LAR of thyroid cancer were as follows: for head CT, 1.1 per 100,000 for boys and 8.7 per 100,000 for girls; for paranasal sinus CT scans, 0.4 per 100,000 for boys and 2.7 per 100,000 for girls; for chest CT scans, 2.1 per 100,000 for boys and 14.1 per 100,000 for girls. The risk of thyroid cancer was substantially higher for girls than for the boys, and from chest CT scans was higher than that from head or paransal sinus CT scans. *Conclusions*: Chest CT scans caused higher thyroid dose and the LAR of thyroid cancer incidence, compared with paransal sinus or head CT scans. Therefore, physicians should pay more attention to protect the thyroid when children underwent CT scans, especially chest CT scans.

## 1. Introduction

The incidence of thyroid cancer appears to be increasing worldwide [1]. In the USA, for example, there was an increase from 3.6 per 100,000 in 1973 to 8.7 per 100,000 in 2002, *i.e.*, a 2.4-fold increase [2]. In China, the incidence of thyroid cancer was also increasing in recent years, as the annual incidence has increased by 14.51% for females during 2003–2007 [3]. In Tianjin (China), the thyroid cancer incidence for females increased from 1.3 per 100,000 in 1981 to 4.2 per 100,000 in 2001 [4]. In Beijing, a total number of 862 thyroid cancer cases were found in 2006–2007, in comparison with 258 cases in 1998–1999, making it one of the fastest growing cancer types in the last 10 years [5]. In addition to improvements in thyroid cancer detection, this increase may reflect changes in environmental factors, including an increase in medical exposure to ionizing radiation from diagnostic imaging, especially during childhood, which is one of the few established risk factors for thyroid cancer and attributed in large part to the growing use of CT [6,7] . The frequency of CT utilization in pediatric patients has increased exponentially because of the sensitivity of CT scanner images, which is ten-fold higher than that of a conventional X-ray device. CT can also be performed within seconds, so there is no need to sedate very young patients [8]. However, the effective doses of radiation from CT scans may be 5–20 times higher than those from routine conventional radiology [9]. Moreover, the smaller size of children means that the effective dose from a CT scan is usually higher than that received by adults [10]. Children as the most radiosensitive subgroup, the lifetime risk of solid cancer induced by radiation exposure is 2 or 3 times higher than general population [11]. It is also well known that children have a longer life expectancy than adults, which may mean that cancer has a greater opportunity to occur and develop.

There have been several previous studies of the radiation dose received in the thyroid during CT scans and estimates have been made of the cancer risk [12,13,14], with the risk estimation being based on the model risk projection, although the number of study samples was very small in some studies. Recently, Pearce *et al.* [15] found that there was a significant association between the estimated radiation doses resulted from CT scans to red bone marrow and brain in childhood and subsequent incidence of leukemia and brain tumors. Moreover, Mathews *et al.* [16] calculated that after accounting for age, sex and year of birth, the overall cancer incidence for exposed from CT scan was 24% higher than for unexposed people, which was statistically significant. These two large record-linkage epidemiology studies proved an increased cancer risk in the future associated with patients receiving CT scans during childhood.

In China, the CT was introduced into the mainland China in 1972, and quickly experienced a sharp increase by 418 CT scanners per year during 1999–2009 [17,18]. CT scans are very common for pediatric patients, especially for head CT [19], but there are few studies concerning the frequency of CT scans for pediatric patients in China, let alone the cancer risk. Consequently, this study was based on one general hospital in China to estimate the radiation dose in the thyroid attributable to the commonly used CT scans and the thyroid cancer risk in pediatric patients.

## 2. Materials and Methods

### 2.1. Collection of CT Scans

This study was performed at a 649-bed general tertiary academic medical center. We searched the radiology information system (RIS) database for pediatric patients (°15 years old) who experienced at least once CT scan between 1 January 2012 and 31 December 2012. We selected three protocols that may cause higher radiation dose for the thyroid [7,20] and relatively high frequencies of scans that were performed on pediatric patients: paranasal sinus CT, head CT, and chest CT. For each patient in the study, we extracted the relevant information from the RIS database in the hospital. This information included the patient ID (a unique code for a patient in this hospital), gender, date of birth, exposure time, type of examination, the body part exposed and the reason for the scan. Microsoft Excel 2003 was used for database management, data cleaning, and to generate descriptive statistics. We did not use any private information related to patients and this study had no effects on the diagnosis and treatment of patients.

### 2.2. Dose Estimation

The scanning parameters used by the 16 Brilliance (Phillips, Best, Netherlands) scanner were abstracted from Digital Imaging and Communication in Medicine (DICOM) headers, which store the technical parameters of each image obtained by a scanner [21,22]. First, we used the command tool “dcmdump” from DCMTK (the German offis open source projects [23]) which can extract DICOM headers to text format. Then, we wrote a Perl script to abstract patient ID, age, the exposed body part and the parameters including the kilovoltage (kVp), tube current (mA), slice thickness (mm), examination type, date of examination, CTDIvol, DLP(dose-length product) and scan model from that text and transform all information to a table in CSV format which can be imported into Excel 2003. Next, we calculated the average value of each parameter by age groups to estimate the organ dose. However, the detector collimation (mm), rotation time (s) and the scan field of view were the same for each CT protocol. Finally, inputting the according averaged parameters to the ImPACT Patient Dosimetry Calculator [24], which was developed by the United Kingdom’s National Radiation Protection Board and it has been validated to calculate tissue-specific absorbed doses from CT, we can obtain the dose absorbed by the thyroid. This method uses a Monte Carlo technique and computational algorithms to estimate the radiation dose by modeling anthropomorphic photon transport. To account for the size difference between adult and small children, age correction factors provided in the ImPACT spreadsheet were applied to the resulted doses [13,25].

### 2.3. Cancer Risk Estimation for the Chinese Population

We used the latest projected model of radiation-induced cancer risk, which was developed in the National Academies’ Biological Effects of Ionizing Radiation (BEIR) VII Report, to estimate the lifetime attributable risk (LAR) of thyroid cancer incidence [26]. The model is based on pooled analyses of Japanese atomic bomb survivors and other medically exposed cohorts, and it employs a linear non-threshold risk model for low-dose radiation, such as medical X-rays, assuming that the cancer incidence is linear relative to the radiation dose. Additionally, this LAR model assumes a period of 5 years was selected as the minimum latency for solid cancers. [26]

The LAR is defined as the probability of radiation-induced cancers in a population of 100,000 who have been exposed to 100 m*G*y, the equations were from BEIR VII (Equations (1)–(4)), were shown as below:

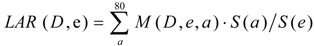
(1)

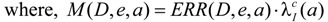
(2)

where, *ERR* /*G_y_* = 0.53 exp[−0.83(*e* − 30) / 10] for males (3)

and *ERR* /*G_y_* = 1.05 exp[−0.83(*e* − 30) / 10] for females (4)

In Equation (1), M (*D*,*e*,*a*) is the excess absolute risk for cancer incidence, e is age at exposure in years, a is attained age in years, S(*a*) is the probability of surviving until age a, the ratio S(*a*) /S(*e*) is the probability of surviving to age *a* conditional on survival to age *e*, *D* is the radiation dose of thyroid in *G*y. In the Equation (2), ERR is from BEIR VII (Table 12-2), the 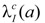 is the baseline incidence rates of sex- and age-specific thyroid cancer in mainland China. In Equations (3) and (4), ERR per *G*y are shown for males and females, respectively.

The LAR of thyroid cancer incidence for pediatric patients in this study was calculated using the baseline thyroid incidence in 2008 provided by National Cancer Center [27] and life table for mainland China in 2008 tabulated by National Bureau of Statistics of China [28].

## 3. Results

### 3.1. Collected Information

Pediatric patients underwent 1,307 CT scans at this tertiary hospital throughout the whole of 2012, and among these 53.3% were head CT (Table 1). The main reasons why most patients received CT scans were trauma and headache. None of them underwent CT scans for suspected thyroid cancer. Among all the scanning protocols, three of them, paranasal sinus CT, head CT and chest CT were thought to perhaps result in higher radiation doses for the thyroid. There were 922 children who received these three CT scan protocols, of which 68% were males; 136 children received paranasal sinus CT scans, 673 children were exposed to head CT scans, and 113 children underwent chest CT scans; among these 39 children experienced repeated CT scans (at least 2 scans during the period of 2012) (Table 2). The highest frequency of repeated CT scans was from 12 year old boy who underwent chest CT scans six times.

**Table 1:** Distribution of CT scans in the hospital in 2012 according to the covered anatomies.

Protocols	Frequency	Percentage (%)
Paranasal sinus	136	10.4
Head	696	53.3
Chest	139	10.6
Abdomen/pelvis	242	18.5
Spine	40	3.1
limb/others	54	4.1
Total	1,307	100

**Table 2:** Demographics of the children in this cohort.

Age (years) at Scan	CT Protocol					
	Paranasal Sinus CT		Head CT		Chest CT	
	Boys	Girls	Boys	Girls	Boys	Girls
0	-	-	5	6	1	1
1–<5	4	-	83	50	6	5
5–<10	35	18	193	82	12	6
10–15	50	29	193	65	51	31
Overall	89	47	470	203	70	43

### 3.2. Radiation Dose

The parameters of different CT protocols for different age groups abstracted from the DICOM headers are shown in Table 3. Most parameters were constant across for pediatric patients in each CT protocol, except for tube current, pitch, kilovoltage, CTDI_vol_ and DLP. We selected the averaged value as the scan parameters for each exposed age group. The typical radiation doses in the thyroid with different CT protocols, by age, are shown in Table 4. The thyroid dose ranges were 1.10–2.45 m*G*y for head CT scans, 0.61–0.92 m*G*y for paranasal sinus CT scans, and 2.63–5.76 m*G*y for chest CT scans.

**Table 3:** Parameters used by three CT protocols considered in this study.

Parameters	Paranasal Sinus CT			Head CT				Chest CT			
Exposed age (years)	1–<5	5–<10	10–15	0	1–<5	5–<10	10–15	0	1–<5	5–<10	10–15
Kilovoltage (kV)	120	120	120	118 ^a^	120	120	120	120	120	120	120
Tube current (mA)	255	237	254	161	178	164	163	313	300	375	330
Rotation time (s)	0.5	0.5	0.5	1.5	1.5	1.5	1.5	0.5	0.5	0.5	0.5
pitch	0.688	0.688	0.688	1	1	1	1	0.688	0.688	0.938	0.938
CTDIvol (m *G*y)	32.4	34.7	35.1	29.9	33.8	33.6	34.1	10.4	11.8	14.1	11.6
DLP (m *G*y·cm)	369.3	337.5	399.0	327.7	374.1	394.2	413.2	243.0	254.2	358.0	353.8
Scan mode	Spiral			Axial				Spiral			
Detector collimation (mm)	16 × 1.5			16 × 1.5				16 × 1.5			
Scan thickness (mm)	5			6				5			
Scan-field-of-view	From frontal sinus to upper alveolar bone			Covered cranium				From thoracic inlet to the costophrenic angle			

Note: ^a^ The assumed kilovoltage from head CT scans for patients in this age group is 120 kV.

**Table 4:** Radiation dose (m*G*y) in the thyroid, by age, using the three CT protocols.

CT protocol	Age (years) at Scan			
	0	1–<5	5–<10	10–15
Paranasal sinus CT	-	0.92	0.65	0.61
Head CT	2.45	1.82	1.38	1.10
Chest CT	5.76	4.34	3.50	2.63

### 3.3. Cancer Risk

Appying the LAR model developed by BEIR VII to the thyroid cancer incidence and life table in China, we estimated the LAR risk for age 0, 5, 10 and 15 years (Table 5). The number of thyroid cancer cases tabulated in the table shown the lifetime risk, assuming 100,000 Chinese children exposed to a radiation dose of 100 m*G*y to the thyroid.

**Table 5:** Lifetime attributable risk of thyroid cancer incidence in the Chinese population.

Gender	Age at Exposure			
	0	5	10	15
Boys	136	91	60	39
Girls	964	639	422	275

Then we applied the risk estimation described above and the current exposure scenario for pediatrics in this hospital, which was characterized in Table 2, to calculate lifetime risk for each of the child patients in the study. We presented the mean and range of LAR by age at exposure and CT protocols in Table 6. 

**Table 6:** Summary of the lifetime attributable risk of thyroid cancer incidence per children based on the different CT protocols in this general hospital.

Age at Exposure	Paranasal Sinus CT	Head CT	Chest CT
	Mean (Min-Max)	Mean (Min-Max)	Mean (Min-Max)
Girls			
0	-	23.6 (-)	55.5 (-)
1–<5	-	14.5 (12.6–48.5)	40.3 (30.1–65.4)
5–<10	3.6 (3.0–4.2)	8.0 (6.3–32.5)	26.4 (16.0–44.7)
10–15	2.2 (1.7–2.6)	3.5 (2.8–4.6)	8.8 (7.0–27.5)
Overall	2.7 (1.7–4.2)	8.7 (2.8–48.5)	14.1 (7.0–65.4)
Boys			
0	-	3.4 (-)	7.8 (-)
1–<5	1.0 (0.9–1.1)	2.0 (1.8–3.6)	5.8 (4.3–8.5)
5–<10	0.5 (0.4–0.6)	1.1 (0.9–3.2)	2.9 (2.3–5.4)
10–15	0.3 (0.2–0.4)	0.5 (0.4–1.3)	1.5 (1.0–7.9)
Overall	0.4 (0.2–1.1)	1.1 (0.4–3.6)	2.2 (1.0–8.5)

Note: The units are the additional number of cancer cases in the lifetime of 100,000 children in China who received CT scans in 2012.

For example, for a 5-year-old child who underwent head CT once, the LAR of thyroid cancer incidence was 8.8 per 100,000 for girls and 1.3 per 100,000 for boys; for chest CT, the LAR was 22.4 per 100,000 for girls and 3.2 per 100,000 for boys; the range of LAR for patients who aged ≥5 years and <10 years received chest CT was from 16.0 per 100,000 to 44.7 per 100,000, this wide range may due to the distribution of exposed age for patients and repeated CT scans. Overall, the LAR of thyroid cancer incidence for boys and girls respectively, was 1.1 per 100,000 and 8.7 per 100,000 for head CT scans, 0.4 per 100,000 and 2.7 per 100,000 for paranasal sinus CT scans, and 2.1 per 100,000 and 14.1 per 100,000 for chest CT scans.

## 4. Discussion

In this study, we estimated the radiation doses received by pediatric patients with the three most commonly used different CT protocols at one hospital by abstracting the scanning parameters from the DICOM headers and we estimated LAR of thyroid cancer incidence using the BEIR VII model based on the Chinese population. The dose and risk estimates provide valuable information, which warn us to be more concerned about potential cancer risk from CT scans for pediatric patients and radiation protection. Compared with the baseline lifetime thyroid cancer incidence for newborn boys and girls in China which are 180 per 100,000 and 580 per 100,000, respectively [29], the higher LAR of thyroid cancer incidence induced by chest CT were 7.8 per 100,000 for boys and 55.5 per 100,000 for girls, were rather lower. Meanwhile, the most exposed body part was head (nearly 64%), next was abdomen (19%), and the third was chest (11%) in this study, which was similar with the result of a survey from Switzerland [30]. The total frequency of CT protocols that may cause high radiation dose for thyroid was very large, so even with a rather lower LAR, we cannot neglect the potential thyroid cancer risk from CT scans.

The radiation doses received in the thyroid varied with different CT protocols. The data showed clearly that the thyroid dose with chest CT scans was higher than that with paranasal sinus or head CT scans, and the results were similar to the published reports. In UK, based on a series of hybrid computational human phantoms coupled with Monte Carlo radiation transport, the thyroid dose from head and chest scans were 2.6 m*G*y and 26.7 m*G*y, respectively, for a newborn baby, 0.5 m*G*y and 21.8 m*G*y for 1-year-old children, 0.6 m*G*y and 17.7 m*G*y for 5-year-old children, and 0.8 m*G*y and 20.3 m*G*y for 10-year-old children [7]. In Hong Kong, Feng *et al.* used a 5-year-old anthropomorphic phantom to measure levels of 2.52 m*G*y for head CT scans and 3.40 m*G*y for chest CT scans [20]. Journy *et al.* applied CT-expo software to estimate the thyroid dose was 8 m*G*y with chest CT scans for children aged <1 year and 7 m*G*y for children older than 1 year but younger than 10 years [31]. Through the comparison with these estimated by different methodology, the thyroid dose caused head CT scans was similar in different studies, but for chest CT scans, the radiation dose was relatively lower in this study. That may because the effect of z-axis overscanning which is defined as extra-exposure along the *z*-axis extends beyond the planned scan region was not to be considered in this study, although *z*-axis overscanning may contribute thyroid organ dose particularly in pediatric chest CT (spiral mode). In the previous report [32], the percentage in the effective dose values between axial and helical scans (z overscanning was taken into account) were up to 43% for the head-neck CT, 70% for the chest CT. Therefore, the estimation of thyroid dose from chest and paranasal sinus CT scans was conservative in this study. Additionally, another factor that underestimated the thyroid dose was the automatic exposure control (AEC) which was used by the CT scanner. We selected the average of tube current for each patient, but there was reported that estimating thyroid doses using the average tube current rotation time product values (CTDI_vol_) would have underestimated thyroid doses by 44% from CT scan on neck regions [14], so the influence of AEC for the radiation organ dose should be considered in the future research. The abstracted parameters were no difference among different age groups in this hospital, although many researches stressed that optimizing the parameters of CT scans was one of important factors to radiation dose reduction [33,34], especially for pediatric patients. This indicated that physicians should be trained to reduce the parameters for pediatric patients, thus decreased radiation dose.

Using the projected risk model from the BEIR VII report, we employed the Chinese demographic and cancer incidence data to estimate the LAR of thyroid cancer incidence. In this study, for a 5-year-old child who underwent head CT once, the LAR was 8.8 per 100,000 for girls and 1.3 per 100,000 for boys; for chest CT, the LAR was 22.4 per 100,000 for girls and 3.2 per 100,000 for boys. For each protocol, the LAR of thyroid cancer incidence for girls was several times higher than that for boys, which is mainly due to the higher baseline cancer incidence in female (about 3 times) and the higher radiation-related risk in this model (about 2 times). Making the comparison with the study from Hong Kong, Feng *et al.* [20] reported that the LAR of thyroid cancer incidence was 4 per 100,000 in 5-year-old boys for each chest CT scan and 21 per 100,000 for girls, as well as 3 per 100,000 for boys from each head CT scan and 15 per 100,000 for girls, we also found that LAR estimated for Chinese girls was higher than Hong Kong girls, while for boys was lower than Hong Kong, which is mainly due to the differences in life table and cancer statistics. The LAR of thyroid cancer incidence decreased with the growth of exposed age in this study, as same with the report that the gender-averaged LAR incidence of thyroid cancer from chest CT scans, from birth to age 100 per 100,000 children, was 46 for 1-month-old children, 43 for 1-year-old children, 31 for 5-year-old children, and 20 for 10-year-old children [31].

There were some limitations in this study. First, this study was only based on one single general hospital in China and one CT scanner, although this situation was common in most hospitals in China, and the more comprehensive study including multi-institution and more CT scanners should be considered in the future that will be more scientific and convincing. Second, the dose estimation used ImPACT software which was based on the bodies of European patients, not from Chinese ones, which may cause some uncertainty, to some extent. Third, there were also uncertainties in estimating LAR by using the method in the BEIR VII report. For thyroid cancer, the multiplicative model was used, reflecting the international basis of the Ron study [35], so the ERR between these populations and Chinese were same. We made no correction for the value of dose and dose rate effectiveness factor (DDREF). Land *et al.* [36] considered the DDREF and the relative biological effectiveness factor appropriate to medical X-ray should have opposite and approximately equal effects on risk. The International Commission on Radiological Protection [37] recommended a DDREF of 2 and the effectiveness per unit absorbed dose of standard X-rays may be about twice that of high-energy photons. While, BEIR VII report recommends the value of 1.5 and the value of relative biological effectiveness (RBE) is still an open question, as the general reference value is 2 or 3. Thus, the LAR of thyroid cancer incidence for patients may be underestimated by at least 30% in our study, if 1.5 for DDREF and 2 for RBE are appropriate.

## 5. Conclusions

In summary, we applied the cancer statistics data and life tabled for China combined with the BEIR VII model to estimate the thyroid cancer risk of pediatric patients who underwent commonly used CT scans. Through this study, it was found that chest CT scans caused higher LAR of thyroid cancer incidence, compared with head CT scans. Given the expansion of CT use in hospitals and institutions, especially for pediatric patients, large number of people exposed means that even small individual risks could translate into a considerable number of cancer incidences, and the study reminds us that radiation doses from CT scans ought to be kept as low as possible and alternative procedures, which do not involve ionizing radiation, should be considered if appropriate. Meanwhile, physicians should pay more attention to the radiation protection of the radiosensitive organs of pediatric patients like thyroid, especially for chest CT scans.
